# Time to act for reappraising the educational system for universal access to opioid analgesics, for quality palliative care and cancer-related pain relief in East Asian countries

**DOI:** 10.1016/j.lanwpc.2021.100270

**Published:** 2021-09-17

**Authors:** Masahiko Sumitani

**Affiliations:** Department of Pain and Palliative Medicine, The University of Tokyo Hospital, Tokyo, Japan

Pain is one of the most common symptoms among patients with cancer. Pain significantly debilitates cancer patients’ health-related quality of life (QOL), and it can persist as one of the independent prognostic factors for survival. When the World Health Organization (WHO) recently reformed the International Classification of Diseases, “cancer-related pain” was appended to be defined as chronic pain attributable to either the cancer itself or its treatments. Opioid analgesics are critical to the effective relief of cancer-related pain. Opioid analgesics were originally recommended by the WHO in 1986 as part of the cancer pain relief guidelines for improving QOL among patients with cancer. Further, the WHO in 2018, extended the indications for strong opioids from conventional moderate-to-severe cancer-related pain to include mild-to-moderate cancer-related pain. Thus, the administration of opioid analgesics has persisted as a mainstay analgesic therapy for cancer-related pain. To expand universal access to effective palliative care and cancer-related pain relief, the problem of supply has been identified as one of the barriers for adequate opioid availability by the International Narcotics Control Board.^1^

In *The Lancet Regional Health – Western Pacific*, Wu and colleagues^2^ report important findings from a retrospective, nationwide database study investigating annual opioid prescription patterns from 2012 to 2017, in which internationally-recommended strong opioids such as oxycodone and hydromorphone were introduced in Taiwan in late 2014. Prior to the introduction of these strong opioids, morphine and transdermal fentanyl were only available as guideline-recommended strong opioids in Taiwan. The authors demonstrate that use of both strong opioids (transdermal fentanyl and morphine) and weak opioids (tramadol, buprenorphine, and codeine) decreased from 2015 with the introduction of oxycodone and hydromorphone. The ratio of strong opioids increased, but that of weak opioids decreased during the study periods. However, the annual cumulative opioid dosages per patient decreased between 2012 and 2017, and the proportion of opioid use among patients with cancer appeared to show a very gradual decline.

Their previous study^3^ of a nationwide survey of cancer patients in Taiwan, conducted in 2014, clearly reported that one-third of patients with cancer-related pain conveyed dissatisfaction with pain control and prescribed analgesics, leading to significantly poor QOL-related outcomes. It should be considered that the under-use and stigma towards opioids in Taiwan and other East Asian countries still present major challenges for cancer pain management. For example, in Japan, the total consumption of opioid analgesics could not still reach the WHO's Adequacy of Consumption Measure (ACM) in early 2010s, which is a globally accepted index used to assess opioid availability for cancer-related pain by the WHO.^4^^,^^5^ Similar to Taiwan, the adequacy of opioid consumption in Japan slightly decreased from 2013 to 2015. This trend would follow that of the United States of America (US), where the 2016 US Center for Disease Control and Prevention (CDC) guidelines recommend restricted use of opioid analgesics for chronic pain, including cancer-related pain, to control the opioid epidemic and crisis.^6^ This US trend clearly demonstrated poor outcomes for cancer pain management among terminally-ill cancer patients.^6^ Hence, conflicting with the CDC guidelines, the latest US government pain management guidelines (Pain Management Best Practices Inter-Agency Task Force, US Department of Health and Human Services, 2020) emphasize the necessity of opioid analgesics for patients with cancer, as well as the risks associated with opioid analgesics. The WHO ACM for opioids has been designed for terminally-ill cancer patients who have a poor prognosis.^5^ Health care professionals and governments in East Asian countries, where the global standard of adequacy of opioid availability has not been achieved and a relatively higher ratio of opioid consumption to annual cancer-related deaths has not been maintained, should recognize that they have not being burdened by an opioid epidemic and crisis in the nations and region.

The present study determines that conventional opioids were merely replaced by newly launched strong opioids, suggesting that resolving supply problems alone was not sufficient to improve adequate opioid availability. In fact, lack of training and awareness of healthcare professionals has been identified as the primary barrier for adequate opioid availability.^1^ Education on balancing the advantages of improving adequate opioid availability with opioid crisis prevention is essential to realize the benefits of opioid analgesics and minimize their drawbacks for the purpose of improving QOL among not only the terminally-ill with pain due to cancer itself, but also patients with cancer experiencing treatment-related pain.^7^ In East Asian countries, governments, scientific associations, healthcare authorities, and individual healthcare professionals should reappraise the specialised nature of their educational system to include the practical use of opioid analgesics and expand universal access to quality palliative care and cancer-related pain relief.

## Declaration of competing interest

The author received funding from Shionogi & Co. Ltd., Nippon Zoki Pharmaceutical, Heartfelt Inc., Nipro Corporation, Eisai Co. Ltd., and Fureasu Co. Ltd; payments from Daiichi-Sankyo, Shionogi & Co. Ltd., Nippon Zoki Pharmaceutical, Mundipharma K.K., Eisai Co. Ltd., MSD, and GlaxoSmithKline K.K.
